# Critical power: An important tool for exercise prescription and the assessment of physiological function

**DOI:** 10.1113/EP092258

**Published:** 2025-01-22

**Authors:** Daniel Muniz Pumares, Samuel Meyler

**Affiliations:** ^1^ School of Life and Medical Sciences University of Hertfordshire Hatfield UK

**Keywords:** exercise prescription, exercise training, fitness testing, maximum oxygen uptake

## 1 INTRODUCTION

Connections link a sequence of three related research papers. The central article which links the other two papers has been published in Experimental Physiology. In a Connections article, an author (or authors) of the central article outlines its principal novel findings, tracing how they were influenced by the first article and how the central article has contributed to the developments made in the third article. The author(s) may also speculate on the direction of future research in the field. Connections articles aim to set the research in a wide context.

The maximum oxygen uptake (${\dot{V}}_{O_{2^{\max}}}$) is the gold standard measure of cardiorespiratory fitness, as it represents the upper limit of oxygen transport and utilisation during strenuous exercise. Indeed, ${\dot{V}}_{O_{2^{\max}}}$ has long been considered a strong predictor of endurance performance, but also mortality and morbidity and an overall marker of health, perhaps more so than other traditional risk factors such as smoking, obesity and type 2 diabetes. Fortunately, ${\dot{V}}_{O_{2^{\max}}}$ is not fixed, and endurance training has consistently been shown to increase ${\dot{V}}_{O_{2^{\max}}}$. However, changes in ${\dot{V}}_{O_{2^{\max}}}$ with endurance training exhibit large inter‐individual variability, even when individuals undertake the same training programme. In the first of this series of connecting papers, Williams et al. (2019) investigated whether different approaches to endurance training affected the magnitude of the response in ${\dot{V}}_{O_{2^{\max}}}$. The authors analysed data from 18 training interventions and 677 participants, whereby individuals undertook high‐intensity interval training, sprint interval training, or moderate‐intensity continuous training. The authors reported that high‐intensity interval training resulted in the greatest increases in ${\dot{V}}_{O_{2^{\max}}}$, as well as the greatest proportion of individuals demonstrating an increase in ${\dot{V}}_{O_{2^{\max}}}$ beyond a predefined change threshold, comprising the technical error of measurement and the minimal clinically important difference of one metabolic equivalent task (1 MET, 3.5 mL kg^−1^ min^−1^).

The study by Williams et al. (2019) illustrates how the intensity of exercise is a key factor underpinning the efficacy of endurance training. Importantly, whilst different constructs have been proposed, a unified approach to quantify and prescribe the intensity of exercise remains elusive and debated (Jamnick et al., 2020). Nonetheless, a popular framework is to prescribe intensity according to three discrete exercise domains: moderate, heavy, and severe, as physiological responses to exercise and the level of metabolic perturbation are similar when exercising within, but not between, each exercise intensity domain (Jamnick et al., 2020).

Notably, the boundaries between exercise domains are demarcated by exercise ‘thresholds’: the gas exchange threshold or lactate threshold, which delineates moderate and heavy exercise domains, and critical power (CP), which represents the boundary between the heavy and severe exercise domains. Despite this, exercise intensity is often prescribed relative to ‘traditional’ maximal anchors, such as a percentage of ${\dot{V}}_{O_{2^{\max}}}$ or maximum heart rate. This approach fails to consider exercise domains, which, among individuals, may be positioned differently in relation to these maximal parameters. Indeed, in the data compiled by Williams et al. (2019), the majority of studies prescribed exercise intensity this way and only ∼13% of the participants took part in a training programme where exercise intensity was anchored to a physiological threshold.

Meyler et al. (2023) tested the consequences of this in the central paper of this connections article. They hypothesised that prescribing exercise relative to traditional anchors of exercise intensity, such as a percentage of ${\dot{V}}_{O_{2^{\max}}}$, would result in higher inter‐individual variability in acute physiological responses to exercise, compared to when intensity was prescribed relative to a physiological threshold. The results demonstrated that, for high‐intensity exercise, prescribing the intensity relative to CP, which represents the highest intensity at which a metabolic steady state may be attained, resulted in less inter‐individual variability and consistent exercise tolerance among participants. To put this finding into perspective, two individuals performing a training session at 75% ${\dot{V}}_{O_{2^{\max}}}$ may experience two very different levels of physiological stress. One individual might be exercising below their CP, which results in sustainable exercise in the heavy domain; however, the other individual might be exercising above their CP, and therefore in the severe intensity exercise, and thus experiencing a much greater metabolic perturbation which elicits ${\dot{V}}_{O_{2^{\max}}}$ and, ultimately, results in task failure. The results presented by Meyler et al. (2023) demonstrate that, where possible, high‐intensity exercise should be prescribed relative to CP, so that exercise domains are accounted for, and all participants experience a similar level of physiological stress.

It is plausible that such a reduction in the variability of acute physiological responses to exercise might result in an analogous reduction in variability and/or a greater magnitude of ${\dot{V}}_{O_{2^{\max}}}$ changes following endurance training. This was the question of a recent meta‐analysis by Meyler et al. (2025), which explored the effect of using physiological thresholds to prescribe exercise training on both the magnitude and variability of changes in ${\dot{V}}_{O_{2^{\max}}}$. The authors collected individual participants’ data from four exercise‐matched studies (139 participants), in which two groups of participants were prescribed exercise relative to a traditional maximal anchor or a physiological threshold in otherwise identical training programmes. Additionally, the authors collected individual participant data from studies prescribing exercise training either relative to a maximal anchor (25 studies and 1190 individuals) or relative to a physiological threshold (18 studies and 354 individuals). Whilst there was no observable difference regarding the variability of ${\dot{V}}_{O_{2^{\max}}}$ changes to training, the results from this meta‐analysis demonstrated higher response rates when exercise was prescribed relative to physiological thresholds. Specifically, 64% of participants increased ${\dot{V}}_{O_{2^{\max}}}$ beyond a clinically relevant threshold of 1 MET when intensity was prescribed relative to physiological thresholds, compared to 16% when intensity was prescribed using traditional anchors (Meyler et al., 2025). This recent meta‐analysis suggests further work is needed to establish how physiological thresholds, and in particular CP, can be applied to design effective training interventions in different groups, and particularly in clinical populations.

**Connected Articles**

- Williams, C. J., Gurd, B. J., Bonafiglia, J. T., … Coombes, J. S. (2019). A multi‐center comparison of VO_2_ peak trainability between interval training and moderate intensity continuous training. *Frontiers in Physiology*, 10, 19.
- Meyler, S., Bottoms, L., Wellsted, D., Muniz‐Pumares, D. (2023). Variability in exercise tolerance and physiological responses to exercise prescribed relative to physiological thresholds and to maximum oxygen uptake. *Experimental Physiology*, 108(4), 581–594.
- Dorff, A., Bradford, C, Hunsaker, A., Atkinson, J., Rhees, J., Leach, O. K., & Gifford, J. R. (2024). Vascular dysfunction and the age‐related decline in critical power. *Experimental Physiology*, 109(2), 240–254.

Markers of health, cardiorespiratory fitness (i.e., ${\dot{V}}_{O_{2^{\max}}}$) and exercise tolerance (i.e., CP) tend to decline with ageing, which is partially explained by the loss of muscle mass. In the third paper of this series, Dorff et al. (2024) investigated whether the age‐related decline in CP was explained not only by muscle quantity (mass), but also by muscle quality, which was assessed as vascular function during single‐leg knee‐extension. The authors reported lower CP in older (∼60 years), compared to younger (∼20 years) healthy adults, irrespective of whether CP was expressed in absolute terms or relative to muscle mass. Strong relationships between CP and different markers of vascular function were also noted. These data suggest that CP may also be sensitive to vascular function. Thus, the findings by Dorff et al. (2024) add to the growing body of evidence suggesting CP integrates several physiological functions, which in turn highlights the role of CP as a physiological variable central to both health and endurance performance (Poole et al., 2021). In other words, whilst ${\dot{V}}_{O_{2^{\max}}}$ remains a ubiquitous concept in exercise physiology, representing the upper limit of the pulmonary, cardiovascular and respiratory systems to transport and utilise O_2_ during exercise involving a large muscle mass, CP is increasingly recognised as a potent marker of physiological function.

Overall, these connecting papers illustrate how ${\dot{V}}_{O_{2^{\max}}}$, a ubiquitous parameter in exercise physiology and the gold standard measure of cardiorespiratory fitness, can be increased with training. However, there is large variability in ${\dot{V}}_{O_{2^{\max}}}$ changes following endurance training, and a key factor explaining such variability is the intensity of exercise (Williams et al., 2019). Prescribing the intensity of exercise remains problematic, and different frameworks have been suggested. However, using physiological thresholds, and particularly using CP to prescribe high‐intensity exercise, has been shown to reduce response variability, which results in a more consistent exercise session among individuals (Meyler et al., 2023). Repeated over time, exercise sessions where the intensity is prescribed relative to physiological thresholds appear to have an impact on the effectiveness of endurance training at improving fitness outcomes, such as ${\dot{V}}_{O_{2^{\max}}}$. Indeed, endurance training programmes are often assessed on their ability to increase ${\dot{V}}_{O_{2^{\max}}}$ (Williams et al., 2019). However, CP may integrate a wide range of physiological systems—possibly wider than ${\dot{V}}_{O_{2^{\max}}}$, with potential to assess physiological function across different systems (e.g., vascular function, Dorff et al., 2024). Combined, these studies highlight the potential and imperative of CP to become a central construct in exercise physiology: a useful tool to standardised exercise prescription and a powerful marker of physiological function in health and disease.

## AUTHOR CONTRIBUTIONS

Both authors have read and approved the final version of this manuscript and agree to be accountable for all aspects of the work in ensuring that questions related to the accuracy or integrity of any part of the work are appropriately investigated and resolved. All persons designated as authors qualify for authorship, and all those who qualify for authorship are listed.

## CONFLICT OF INTEREST

None declared.

## FUNDING INFORMATION

None.
